# Necrotizing Fasciitis After Herpes Zoster Infection: A Rare Case With Diagnostic Difficulties

**DOI:** 10.7759/cureus.24805

**Published:** 2022-05-07

**Authors:** Bilgen Can, Betül Gözel

**Affiliations:** 1 Plastic and Reconstructive Surgery, Balıkesir University Hospital, Balıkesir, TUR; 2 Plastic Reconstructive and Aesthetic Surgery, Balıkesir University Hospital, Balıkesir, TUR

**Keywords:** herpes zoster, lrinec score, urgent debridement, necrotizing fasciitis, shingles

## Abstract

Necrotizing fasciitis is a rare complication of herpes zoster. Because of its rarity, it may be overlooked in the differential diagnosis of patients with bacterial superinfection on herpes zoster lesions.

We present the case of a 59-year-old woman with diabetes mellitus receiving oral antibiotic therapy with the diagnosis of bacterial superinfection due to herpes zoster involving the C7-T8 dermatomes bilaterally. She presented at our emergency department with a deteriorated general condition and signs of sepsis. Her Laboratory Risk Indicator for Necrotizing Fasciitis (LRINEC) score was 10.

Necrotizing fasciitis can arise from herpes zoster lesions. However, its rarity can lead to delayed treatment which can further result in significant morbidity, and even mortality, and should be considered among patients presenting with bacterial superinfections. The LRINEC score is very effective and practical for differentiating necrotizing fasciitis from bacterial superinfections. In case of suspicion, follow-up must be conducted on an inpatient basis.

## Introduction

Herpes zoster infections occur in 10-20% of adults with chickenpox infection during childhood [1]. The reactivation of the virus, which remains latent in the cranial or sensory ganglia, after the primary infection in association with immunosuppression, mechanical, or psychological stress, causes a secondary infection, which manifests as vesicular rash and radicular pain in the affected dermatomal area [2].

Necrotizing fasciitis is a complication of primary varicella infection among both children and adults. According to previous case studies, it can develop at a rate of 1-4% [3-7]. However, to date, only five studies have reported necrotizing fasciitis superimposed on herpes zoster [1,8-11]. Due to its rarity, we aim to draw attention to this condition, in which delayed diagnosis may cause severe morbidity and even mortality, as seen in our case that had delayed diagnosis.

## Case presentation

**Figure 1:**
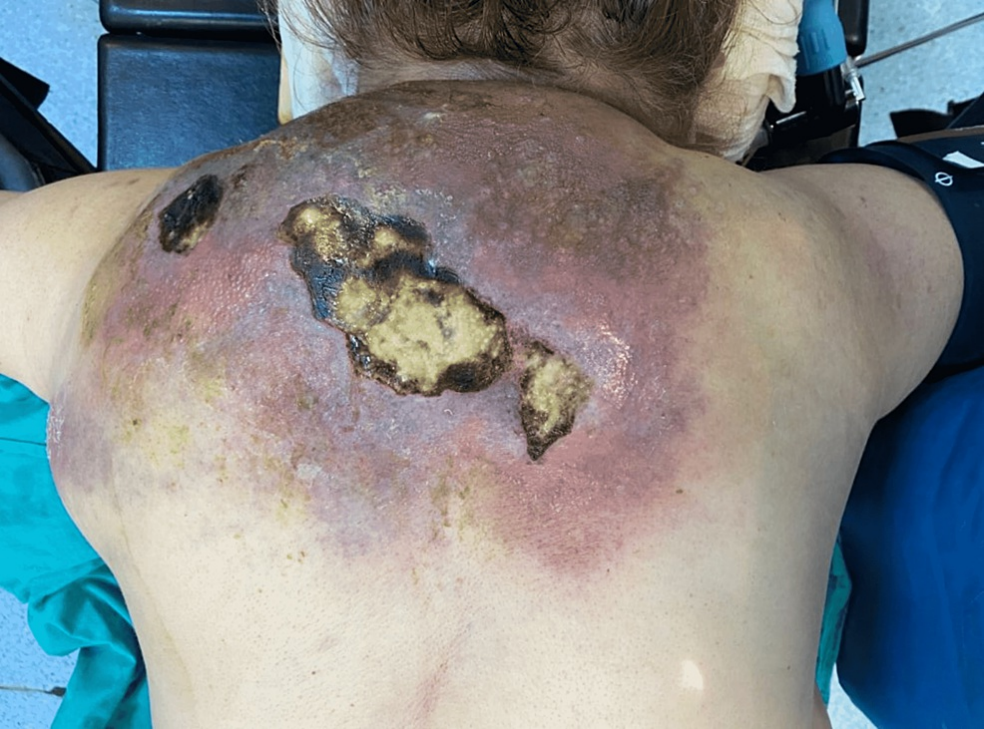
Massive abscess with zona rash and skin necrosis on the back.

A 59-year-old obese woman with diabetes mellitus was diagnosed with bacterial superinfection due to herpes zoster involving the C7-T8 dermatomes bilaterally. Oral antibiotic therapy was prescribed for the bacterial superinfection in addition to oral acyclovir therapy by her general practitioner. For the next 20 days, because her skin lesions progressed, she was referred to the dermatology, infectious diseases, and general surgery departments where oral and topical antibiotics were prescribed without any laboratory testing. At the end of the 20th day, the patient was admitted to our hospital’s Emergency Department with a fever and poor general condition. She had a bilateral diffuse vesicular rash in the C7-T8 dermatomal area on her back, along with induration, fluctuating swelling extending to the entire thoracic back and the left axillary area, and skin necrosis in some areas (Figure 1).

**Figure 2:**
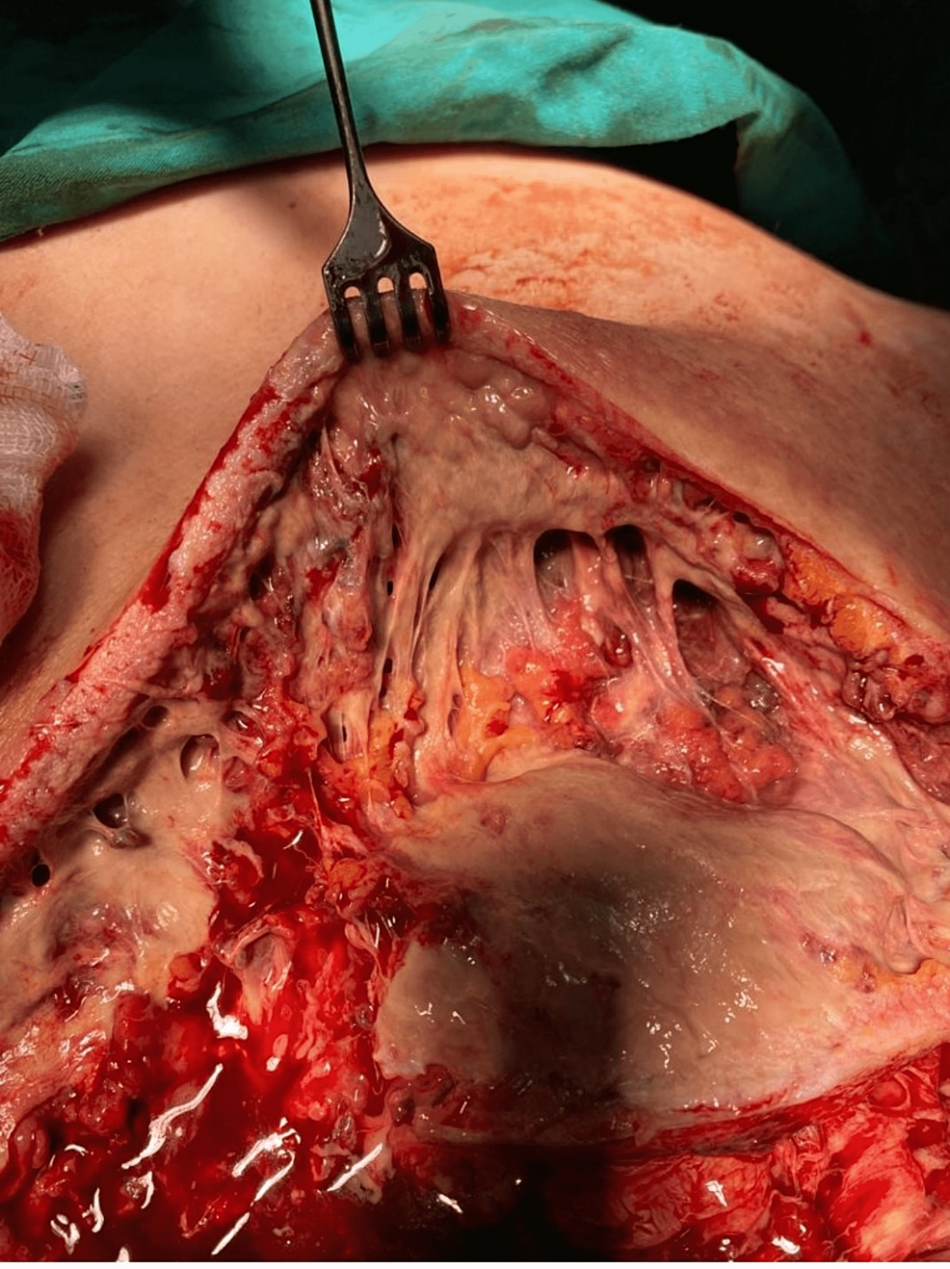
Abscess spread in the subcutaneous planes with soft-tissue necrosis.

On presentation, her blood pressure was 110/55 mmHg, and her heart rate was 113 beats per minute. In addition, the hemogram revealed neutrophil-based leukocytosis and thrombocytosis. She also had moderate creatinine elevation compatible with acute renal failure. C-reactive protein level was 250 mg/dL, sodium was 130 mg/dL, and glucose was 110 mg/dL. The patient was diagnosed with necrotizing fasciitis. Her Laboratory Risk Indicator for Necrotizing Fasciitis (LRINEC) score was 10 which shows a high risk for necrotizing fasciitis. The patient was taken to the operating room. Subcutaneous tissues were explored with the debridement of the necrotic skin. It was observed that the dense abscess extended under the trapezius muscle and caused necrosis in all subcutaneous soft tissues, including the muscle (Figure 2).

**Figure 3:**
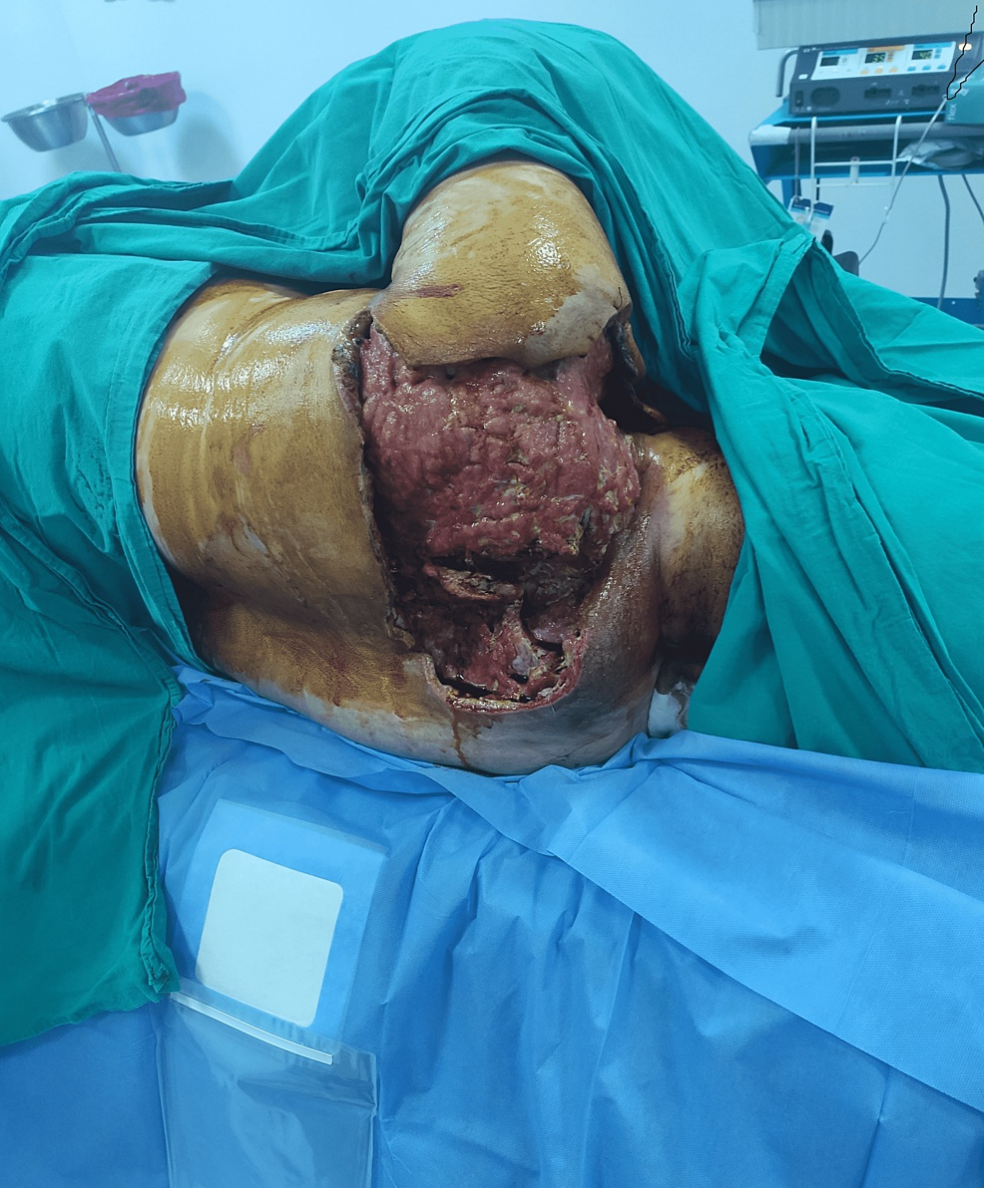
Healthy granulation tissue in the defect site as a result of serial debridement.

**Figure 4:**
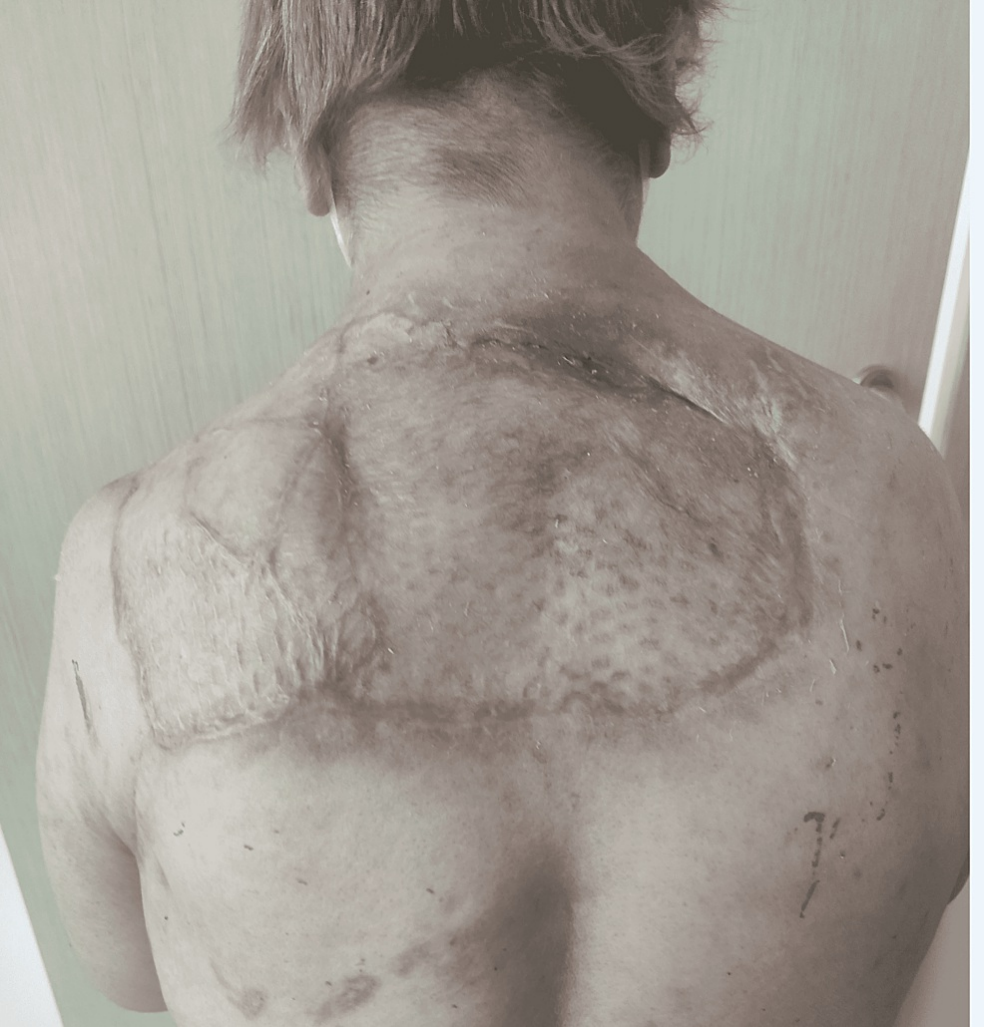
Tissue defects were skin grafted six months after discharge.

The necrotic skin and subcutaneous tissues, fascia, and necrotic muscle were debrided, and deep-tissue cultures were obtained and sent for testing. The patient was followed up in the intensive care unit with hydration support and close monitoring. Debridement was repeated under general anesthesia after 48 hours for a second look. The dressings of the patient, who developed a very large tissue defect in the posterior cervical area, the upper half of the back, and the axillary area, were changed under sedation twice a day. *Streptococcus pyogenes* was identified in the wound samples and she was given carbapenem 500 mg every six hours. After the second debridement, C-reactive protein and leukocyte levels decreased, and her kidney functions returned to normal. After six days of follow-up in the intensive care unit, she was transferred to the clinic where dressing changes continued under sedation twice a day. Healthy granulation tissue was detected in the defect area at the end of the 18th day of her hospitalization, and the patient was ready for reconstruction (Figure 3). Finally, defect areas were skin grafted (Figure 4).

## Discussion

Necrotizing fasciitis is a complication noted among both children and adults after primary varicella-zoster infection. However, only five cases have been reported in the literature on necrotizing fasciitis as a complication of herpes zoster [1,8-11]. Necrotizing fasciitis may not be considered in patients who develop a bacterial superinfection on herpes zoster rashes because of its rarity, but serious morbidity or mortality can occur due to delayed diagnosis.

Although the pathogenesis of necrotizing fasciitis after herpes zoster is poorly understood, theories propose that varicella rashes provide bacterial entry, and while the varicella virus causes a shift in helper T-lymphocytes and reduces cellular immunity, and exotoxins produced by *Streptococcus pyogenes* act as superantigens [7]. Literature supports this theory as in most of the cases presented in the literature, *Streptococcus pyogenes* is the most common organism identified in the wound samples as in our patient. Decreased cellular immunity may also play a role in the delay in diagnosis. Due to the lack of proper cellular reaction to the infection, clinical progress may reveal atypically. Hence, the general condition of the patient and skin lesions should not be the only criteria for diagnosis, and evaluation of laboratory parameters should be performed for every patient with close monitoring. For this purpose, the LRINEC score offers a very simple and useful algorithm [12]. LRINEC, a scoring system developed by Wong et al., uses the leukocyte, hemoglobin, glucose, sodium, creatinine, and C-reactive protein levels, with a 92% positive and 96% negative predictive value for diagnosing necrotizing fasciitis. Hence, hospitalization of patients with moderate LRINEC scores and close monitoring may prevent delay in diagnosis.

When we investigate all the five cases reported in the literature, it is clear that no laboratory test was performed during the first presentation of the patients [1,8-11]. All patients were diagnosed with bacterial superinfection, were prescribed antibiotic therapy, and admitted to the emergency department with the deteriorated general condition after 24-48 hours. considering that two out of five reported patients died, we strongly recommend that laboratory tests should be performed in all patients with bacterial superinfection after herpes zoster, and the LNIREC score must be used in the differential diagnosis with necrotizing fasciitis.

Urgent debridement, intravenous antibiotic therapy, and fluid resuscitation are the three corners of the treatment as in classical necrotizing fasciitis [13]. Fung et al. suggested that because of the intense vascularity of the eyelids, debridement can wait in periorbital necrotizing fasciitis until auto-demarcation appeared. Observation for several days with close monitoring and antibiotic therapy may allow auto-demarcation in periorbital necrotizing fasciitis. However, this is not true for truncal and extremity necrotizing fasciitis which have high mortality rates of 20-50% [10].

Because in all the cases presented in the literature, wound samples revealed *Streptococcus pyogenes*, empirical antibiotic therapy must cover this organism. Sharma et al. suggested that initiation of acyclovir within the first 24 hours when the first rashes appear helps reduce mortality and morbidity from necrotizing fasciitis. However, we presented a case of necrotizing fasciitis after varicella-zoster infection, and there is no clear information about using acyclovir in necrotizing fasciitis after herpes zoster infection. We continued acyclovir until the patient’s vesicular rashes alleviated around 30 days from the first appearance.

## Conclusions

Because of its rarity, necrotizing fasciitis as a complication of herpes zoster infection is overlooked or misdiagnosed as simple bacterial superinfection. Delayed diagnosis may cause severe morbidity due to large tissue defects or even death. Hence, the LRINEC score must be used for all patients who have infectious symptoms of herpetic rashes, and in case of suspicion, patients must be hospitalized under close monitoring.
